# Therapeutic Prospective of Infused Allogenic Cultured Mesenchymal Stem Cells in Traumatic Brain Injury Mice: A Longitudinal Proton Magnetic Resonance Spectroscopy Assessment

**DOI:** 10.5966/sctm.2016-0087

**Published:** 2016-08-08

**Authors:** Sushanta Kumar Mishra, Poonam Rana, Subash Khushu, Gurudutta Gangenahalli

**Affiliations:** ^1^ NMR Research Centre, Institute of Nuclear Medicine and Allied Sciences, Defense Research and Development Organisation, Timarpur, Delhi, India; ^2^ Division of Stem Cell and Gene Therapy Research, Institute of Nuclear Medicine and Allied Sciences, Defence Research and Development Organisation, Timarpur, Delhi, India

**Keywords:** Mesenchymal stem cells, Magnetic resonance imaging, Stem cell therapy, Proton magnetic resonance spectroscopy, Traumatic brain injury

## Abstract

Improved therapeutic assessment of experimental traumatic brain injury (TBI), using mesenchymal stem cells (MSCs), would immensely benefit its therapeutic management. Neurometabolite patterns at injury site, measured with proton magnetic resonance spectroscopy (1H‐MRS) after MSCs transplantation, may serve as a bio‐indicator of the recovery mechanism. This study used in vivo magnetic resonance imaging and 1H‐MRS to evaluate the therapeutic prospects of implanted MSCs at injury site in experimental mice longitudinally up to 21 days. Negative tissue contrast and cytotoxic edema formation were observed in susceptibility‐based contrast (T2*) and an apparent diffusion coefficient map, respectively. Lesion site showed decreased *N*‐acetylaspartate, total choline, *myo*‐inositol, total creatine, glutamate‐glutamine complex, and taurine neurometabolic concentrations by 1H‐MRS investigation. There was a considerable decrease in locomotor activity, depression index, and cognitive index after TBI. It may, therefore, be inferred that MSC transplantation prompted recovery by decreasing negative signals and edema, restoring metabolites to baseline concentrations, and enhancing behavioral activity. Overall findings support the potential of MSC transplantation for the enhancement of endogenous neuroprotective responses, which may provide future clinical applications for translating laboratory research into therapeutic clinical advances. Stem Cells Translational Medicine *2017;6:316–329*

**Significance Statement**

Traumatic brain injury (TBI) is a unique kind of injury and its treatment is a complex clinical issue. An insult to brain directly affects the gene expression profile for protein synthesis and eventually affects metabolic levels. This study provided a detailed description of the effects after homing of stem cells to the lesion area by administering fluorescent‐labeled mesenchymal stem cells (MSCs) and then performing analysis using fluorescent microscopy and serial in vivo proton magnetic resonance spectroscopy using a 7.0 Tesla magnetic resonance imaging (MRI) scanner. After injury, the affected profile of neurometabolites at an injury site, and the neurometabolite profile's subsequent restoration to normal levels detected in the study after stem cell transplantation, revealed a pattern. It was proposed that this likely was a recovery signature (bio‐indicator) of TBI after stem cell therapy. This study was the first to report on the patterned recovery of altered neurometabolites after transplantation of MSCs in a TBI mice model, which possibly occurred as a regenerative response. Behavioral studies conducted simultaneously have also been well‐correlated with MRI findings.

## Introduction

Traumatic brain injury (TBI) is a major universal cause of a broad range of physical, cognitive, behavioral, and emotional disabilities, depending on the type, severity, and location of injury 1. Starting from a primary impact event, it may develop into a lifelong chronic disability, with secondary widespread brain tissue damage 2. Treatment of TBI is a complex clinical issue requiring highly accurate and sensitive diagnosis and is rendered even more difficult by complex intrinsic signaling, lack of pathophysiological biomarkers, and limited self‐recovery ability of the brain 3. To date, there has been no development of clinical therapeutics to effectively interrupt secondary brain injury 4, which occurs progressively in the period following the initial trauma.

Stem cell transplantation offers promising therapeutic potential for various tissue injuries 5. A number of experiments on animal models have reported the beneficial effects of systemic stem cell transplantation after tissue injuries 5 6 7 8. Mesenchymal stem cells (MSCs) are multipotent in nature and have the capacity to differentiate themselves into various mesodermal tissue lineages depending on the microenvironment and signaling processes 9. In addition to their immune regulatory behavior, their paracrine mechanisms involving the release of trophic and immunomodulatory factors 10 would determine the manner in which MSCs are used. MSCs respond to intrinsic signal molecules after being transplanted and homed to the lesion site 11. In the present study, homing at the lesion area was monitored by administering fluorescent‐labeled MSCs and analyzing them with fluorescent microscopy. Complete response of MSCs was determined by their niche, comprising the surrounding cells and the releasing factors of stem cells, which may have either autocrine or paracrine actions 10, 12. Cell‐based treatment of acute TBI is clinically realistic and could facilitate the smooth progress of functional improvement 8, 13.

An insult to brain directly affects the gene expression profile for protein synthesis and eventually affects metabolic levels 14. The alteration in neurometabolites at injury site, and its restoration following stem cell transplantation, may be used as a bio‐indicator during treatment of TBI 15. Most research studies have evaluated stem cell tracking and effects of cell‐based treatment using immunohistochemistry (IHC) and enzyme‐linked immunosorbent assay, which cannot provide longitudinal in vivo information about stem cell dynamics, fate, and synergistic response after transplantation 16, 17. With the objective of gaining a better understanding about these areas under ideal conditions, the resolution of alterations in localized neurometabolites with in vivo proton magnetic resonance spectroscopy (1H‐MRS) was explored. Magnectic resonance spectroscopy is a noninvasive neuroimaging technique that allows the quantitative assessment of metabolites in the selected predefined region, with potential application in preclinical and clinical studies 18 19 20. In several such investigations of mild to severe TBI, 1H‐MRS is predominantly used to specifically identify alterations in neurometabolic profiles 19, 20. Several metabolites detected by 1H‐MRS at the lesion site are highly sensitive to pathophysiological conditions such as inflammation, mitochondrial dysfunction, oxidative stress, bioenergetic alteration, membrane disruption, and hypoxia or ischemia 14, 18 19 20.

Many human TBI reports have used 1H‐MRS and spectroscopic imaging to demonstrate an early decrease (24 hours) in the neural marker *N*‐acetylaspartate (NAA), which persists for 1 week 21 22 23. One investigator observed increased concentration of combined glutamate‐glutamine complex (Glu^−^ + Gln [Glx]) in white matter and decrease in gray matter after 13 days following injury 18, 24. Ashwal et al. noted an elevated myoinositol (mI) level after TBI in children 25. Preclinical TBI models have improved overall understanding of metabolic alterations and supported the interpretation of clinical data. Xu et al. observed a significant reduction in NAA, glutamate (Glu), taurine (Tau), *myo*‐inositol (Ins), and total choline (GPC + phosphocholine [PCh] [tCho]), whereas an increase was seen in glutamine at early time (4 hours) after TBI in rats 20. Lescot et al. demonstrated a reduction in NAA and elevation of lactate (Lac), 24 hours after focal TBI 19. There is no available report on the alteration of neurometabolites after therapeutic efficacy of MSCs in animal models. To the best of our knowledge, this is a first time report on the recovery of altered neurometabolites after MSCs transplantation in TBI mice model in a serial in vivo 1H‐MRS study using a 7.0 Tesla MRI scanner (Bruker BioSpec USR 70/30, AVANCE III; Bruker Corporation, Billerica, MA, https://www.bruker.com).

It is important to link the spectroscopic findings of clinical and preclinical studies, to specific pathological mechanisms like changes in longitudinal (T1) and transverse (T2/T2*) relaxation time at lesion site and histological findings. It is equally essential to show a relationship between these factors and behavioral outcomes in TBI survivors 19, 20, 26. Much evidence is available to illustrate the direct correlation between the mentioned factors and behavioral deficit after TBI, whereas infusion of MSCs in brain injury subjects improves neurological outcomes 5, 15, 17, 27. A behavioral assessment was conducted to support MRI findings and, additionally, to test the therapeutic benefits of MSCs transplantation in TBI mice.

## Materials and Methods

### Selection, Sampling, and Care of Animals

All experimental procedures were conducted under the approval of the animal ethics committee of the Institute of Nuclear Medicine and Allied Sciences; all procedures were carried out in accordance with the standard recommended manual on the use and care of laboratory animals. Eighty one adult healthy BALB/c male mice (25–30 g, 6–8 weeks old) were taken for the experiment. They were given laboratory chow and water ad libitum and maintained under temperature‐controlled conditions (25°C ± 2°C) with 12‐hour light/dark cycles. The mice were randomly distributed into five groups as follows:

- Group 1 (5 mice): This group was used for isolation of stem cells from bone marrow.
- Group 2 (6 mice): This group was utilized for IHC to confirm stem cells homing. Three mice were sacrificed on day 3 (D3), and the remaining 3 on D7 after MSC transplantation.
- Group 3 (20 mice): This group was subjected to simultaneous MRI and brain water content measurement at 5 time points (4 mice per time point).
- Group 4 (20 mice): This group was investigated for longitudinal in vivo 1H‐MRS study at preinjury, after‐injury, and at other different time points, after MSC transplantation in the same mice. Ten mice from this group were assigned for conducting behavioral tests after the final 1H‐MRS study.
- Group 5 (30 mice): To make a comparative assessment of the behavioral test, this group was subdivided into three groups, namely the control group, injury group, and stem cell‐transplanted control group.

**Figure 1:**
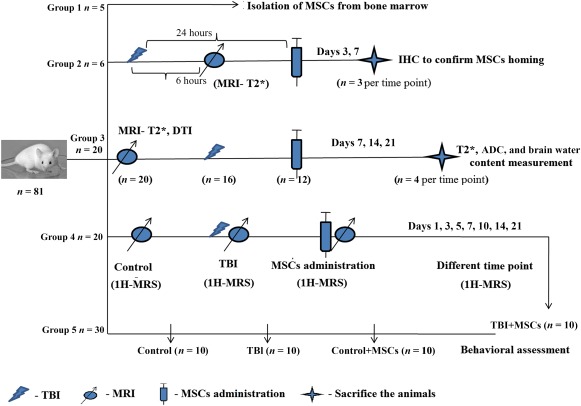
Schematic representation of mice distribution in different groups. Group 1 (5 mice) was used for isolation of stem cells from bone marrow; group 2 (6 mice) was utilized for IHC to confirm stem cell homing. Three mice were sacrificed on D3, and 3 on D7, after MSCs transplantation; group 3 (20 mice) was assigned for simultaneous MRI (T2* and ADC) and brain water content measurement at 5 time points (4 mice per time point); group 4 (20 mice) was investigated for longitudinal in vivo 1H‐MRS study in before injury, after injury, and at different time points after MSCs transplantation in the same mice. Ten mice from this group participated in the behavioral test after final 1H‐MRS study; group 5 (30 mice) was divided into 3 groups (control, injury, and stem cell‐transplanted control groups) to assess behavioral changes with the behavioral test. Abbreviations: ADC, apparent diffusion coefficient; D, day; IHC, immunohistochemistry; 1H‐MRS, proton magnetic resonance spectroscopy; MRI, magnetic resonance imagining; MSC, mesenchymal stem cell; TBI; traumatic brain injury.

A detailed schematic representation of distribution of the experimental mice is presented in Figure 1.

### Stem Cell Isolation and Characterization

MSCs were isolated from the tibia and femur of healthy BALB/c mice (*n* = 5), after cervical dislocation. The protocol for MSC isolation was mainly based on (1) frequent medium change in primary culture, and (2) diminishing the trypsinization time 28, 29. The cells were subsequently cultured in Dulbecco's modified Eagle's medium‐low glucose medium supplemented with 15% fetal bovine serum (FBS; Thermo Fisher Scientific Life Sciences, Waltham, MA, http://www.thermofisher.com), 2 mM/L‐glutamine (Sigma‐Aldrich, St. Louis, MO, http://www.sigmaaldrich.com), and 1% streptomycin/penicillin/amphotericin b (Sigma‐Aldrich). The culture was maintained at 37°C in a humidified atmosphere containing 95% air and 5% CO_2_. The medium was changed twice during the initial 72‐hour period to remove nonadherent red blood cells and macrophages and, thereafter, twice per week. Passaging was carried out by treating with 0.025% trypsin containing 0.02% EDTA, for 2–3 minutes at room temperature. All experiments were performed using cells from fourth passage.

Approximately 2 × 10^5^ cells were selected for the determination of surface antigens of stem cells by immunocytochemistry. The cells were stained with fluorescent isothiocyanate (FITC)‐conjugated rat anti‐mouse CD34, CD45, CD11b, Sca‐1, and CD90.2 (Thy1.2) (BD Biosciences, San Diego, CA, http://www.bdbiosciences.com), at a dilution of 1:250 in phosphate‐buffered saline (PBS) at 4°C for 60 minutes. The monolayer cells were washed with 1× PBS, nuclear stained with Hoechst 33342, and fixed in 2% paraformaldehyde. After washing in PBS, images were captured using a fluorescent microscope. Differential assays like osteogenic and adipogenic lineages were examined in MSCs 28, 29. The confluent cultured cells were incubated in osteogenic and adipogenic conditioned media. The induction medium was changed on alternate days for a period of 21 days, following which the cells were fixed and stained with 2% Alizarin Red S and 0.5% Oil Red O for 5 minutes, to detect osteogenesis and adipogenesis, respectively.

### Weight Drop Injury Model

Traumatic brain injury was induced in mice as described by Marmarou's weight‐drop model with some modifications, which closely mimics the closed head injury 1, 30, 31. Mice were anesthetized with a cocktail of ketamine (80 mg/kg b/w) and xylazine (10 mg/kg b/w) and placed onto the stereotactic holder under the weight‐drop device. A circular steel helmet was placed on the mouse head. A cylindrical brass of weight (35 g) was dropped freely from a height of 40 cm on the steel helmet, with an approximate induced force of 0.137 newtons, to create a diffuse type of injury. After injury, the animals were monitored for 30 minutes with supplemental O_2_ and returned to their respective cages until MRI assessment. The occurrence of injury was confirmed in the MRI scan taken after 6 hours after injury in all the mice used for TBI.

### Fluorescent Labeling and Cell Transplantation Procedure

PKH26 is a red fluorescent dye, which mainly binds to the cell membrane. It has been used as a cell tracer to locate cells after transplantation in host for a long time 13. MSCs from the fourth passage were collected and labeled with red fluorescent dye PKH26 (Sigma‐Aldrich), according to the manufacturer's protocol. Briefly, MSCs were washed by a serum‐free medium, and resuspended in 500 µl of dilution buffer provided in the manufacturer's labeling kit. The cell suspension was mixed with an equal volume of the labeling solution containing 4 × 10^−6^ M PKH26 in the dilution buffer and incubated for 5 minutes at room temperature. The reaction was arrested by adding 1 ml FBS, centrifuged at 300*g* for 5 minutes. To completely remove excess dye, the cells were dissolved with 1× PBS and washed three times in PBS. The treated cells were counted and a total of 1.25 × 10^6^ MSCs were suspended in 200 µl of PBS for transplantation. An identical number of MSCs (1.25 × 10^6^ per mouse) was administered intravenously into the tail vein of each TBI mouse (24 hours after injury), with the help of a 26‐Gz insulin syringe. No immunosuppressant was used in this study as MSCs are hypoimmunogenic in nature.

### Magnetic Resonance Imaging and 1H‐MRS Acquisition

All MRI experiments were carried out on 7T horizontal bore animal MRI scanner (Bruker BioSpec USR 70/30, AVANCE III; Bruker Corporation, Billerica, MA, https://www.bruker.com), equipped with a BGA12S gradient system connected with a Bruker Paravision 5.1 console (Bruker). Animals were anesthetized using ketamine and xylazine cocktail, and they were placed in prone position on an animal bed and then slid into the center of the magnet bore. Radio frequency excitation was accomplished with a 72‐mm inner diameter linear birdcage coil. A mouse brain array coil (single tuned 1H, 2 × 2 array, four channels) was used for signal reception.

MRI and 1H‐MRS scans were performed on the same group of control mice before and after TBI. Further scans were performed on the same TBI mice after administration of stem cells on D1, D3, D5, D7, D10, D14, and D21 in the post‐TBI period.

A three‐orientation (axial, sagittal, and coronal) scout image using a fast, low‐angle single shot sequence was obtained to localize the mouse brain. Multislice axial images were acquired to cover the entire brain (slice thickness: 0.7 mm; number of slices: 11; field of view: 2.5 × 2.5 cm^2^; matrix size: 256 × 256). For localization and identification of anatomical landmarks, a rapid acquisition with relaxation enhancement sequence (TurboRARE sequence; echo time [TE]: 26 ms; repetition time [TR]: 2,500 ms; number of averages: 1) was used to acquire T2‐weighted images of the mouse brain. First‐ and second‐order localized voxel shimming were performed with the fast automatic shimming technique mapping along with projections (FASTMAP), and a full‐width half‐maximum line width of water signal ≤15 Hz. was achieved. Shimming was frequently found to be more challenging in the injured area, mainly due to local field inhomogeneities. If repeated shimming at injury site could not achieve a line width of ≤15 Hz, then those spectra were discarded.

Localized proton spectra images were acquired using a point resolved spectroscopy sequence (PRESS). The voxel size of 1.5 × 2 × 2 mm^3^, 6 µl was placed within the region of interest (injury area), with the maximum volume containing tissue only from the intended structure, thus minimizing the contribution from surrounding tissue, as well as from partial volume effects. The following scan parameters were used for the PRESS spectra: TR/TE: 2,500/20 ms; spectral width: 4 kHz; 2,048 complex data points; and 512 signal averages with total acquisition time of 21 minutes. Water suppression was performed using variable power radiofrequency pulses with optimized relaxation delay (VAPOR). A spectrum without water suppression was acquired using identical parameters with 16 average scans, for the purpose of quantitative analysis. Eddy current compensation and static magnetic field drift correction were applied during data acquisition.

T2* maps were acquired using multigradient echo (MGE)_T2* sequence (TR: 1500 ms; 12 echos TE/echo spacing: 4.50–65.45 ms/5.54 ms; matrix size: 256/192; flip angle: 30°) to study the relaxation time alteration in injury area. Apparent diffusion coefficient (ADC) maps were acquired using diffusion tensor imaging (DTI) sequence (TR/TE: 3800/31 ms; number of diffusion encoding directions: 46; b value: 670 seconds per mm^2^; matrix size: 128/128). All other parameters were kept similar for MGE_T2* and DTI sequences (field of view: 2.5 cm; slice thickness: 0.7 mm; and number of slices: 15).

### Spectra Processing

All spectra were preprocessed using Topspin 2.1 (Bruker) for baseline correction 32. For quantitative assessment of brain metabolites, the MRS raw data were analyzed using the linear combination (LC) model, which uses a basic model of spectra, acquired from in vitro samples of pure chemicals, to estimate in vivo neurometabolic concentrations and the unsupressed water signal from the prescribed voxel, as a reference for each scan, to enable correction of small variations in coil sensitivity. Each spectrum was fitted to a set of known spectra from a series of metabolite solutions with known concentrations 14, 32. Spectral peaks were assigned with reference to the water peak (4.82 ppm). The LC‐model automatically calculates metabolite concentrations and uncertainties, using Cramér‐Rao lower bound formalism. An estimate was considered as relevant when the corresponding bound was below 20% and the estimated standard deviation (%SD) of the metabolite fit, as output from LC model, to ensure reliable metabolite quantitation. Relative concentrations of all observed metabolites and macromolecules were calculated for further analysis in terms of institutional units (IU). Neurometabolites are abbreviated as follows: Cr, creatine; PCr, phosphocreatine; GABA, γ‐aminobutyric acid; Gln, glutamine; Glu, glutamate; Ins, *myo*‐inositol; Lac, lactate; MM, macromolecules; NAA, *N*‐acetylaspartate; NAAG, *N*‐acetylaspartatyl glutamate; GPC, glycerophosphocholine; PCh, phosphocholine; Tau, taurine; tCr, Cr^+^PCr; tCho, GPC^+^PCh; Glx, Glu^–^+Gln.

### T2* Relaxation Time Measurement

T2* relaxation times of injured regions in mice brain were calculated using Bruker's Paravision 5.1 software , to place the region of interest (ROI) with the help of the image sequence analysis (ISA) tool 29. ROIs (area, 0.0211 cm^2^) were drawn at the site of injury in three consecutive slices with three ROIs per slice. T2* relaxation times (msec) were calculated by fitting the signal decay curve of multiecho data, using biexponential curve fitting, as per the manufacturer's instructions. Variation in the calculated values was seen due to variation in MRI bias field, variability in animal and coil positioning during imaging. Thus, absolute bias correction was needed in ISA tool to measure the exact relaxation time at specified ROIs by normalized T2* values.

### Water Content Measurement in Brain

The brains of five mice in every group were examined for presence and amount of water content after imaging at regular and specific time points as described earlier 30, 31. In brief, the selected mice were sacrificed at different time points, brains removed and the two hemispheres dissected out on ice. Coronal sections of the injured hemisphere (4.0 mm) were separated and wet weight (WW) recorded. Samples were then heated at 100°C for 24 hours and measured for dry weight (DW). Water content was calculated and expressed as a percentage ([(WW − DW)/WW] × 100). A comparison was made of these values obtained in control animals (pre‐TBI) with those animals after injury (TBI animals) and at subsequent three time points (D7, D14, D21 after transplantation).

### Immunohistochemistry

Confirmation of proper homing of fluorescent labeled stem cells to the injury site after systemic transplantation was achieved by histological analysis of six mice on D3 and D7 after transplantation. Intracardiac perfusions were carried out with 20 ml of 4% paraformaldehyde in phosphate buffer saline (pH, 7.4), on animals sacrificed on D3 and D7 after transplantation. Brains from individual mice were dissected out, immersed overnight in fixative at 4°C, and kept in 10% formaldehyde solution until use. The preserved tissue samples were embedded in paraffin wax blocks and then sliced into 6 µm thick coronal sections with a cryotome. Sections were deparaffinized with xylene for 5 minutes and dehydrated by gradations of ethyl alcohol. Endogenous peroxidase was quenched for 10 minutes with 30% H_2_O_2_ in absolute CH_3_OH (1:9). The brain sections were then rinsed 3 times with PBS and incubated for 10 minutes in denaturing and blocking solutions. Sections were then stained with Hoechst 33342 for 5 minutes at room temperature and were thoroughly washed with 1 × PBS. The images were captured in fluorescent microscope (PKH26: Ex/Em‐ 551/567 nm; Hoechst 33342: Ex/Em‐ 361/497 nm) to visualize stem cells homed at the lesion area 13.

### Behavioral Assessment

All behavioral studies were conducted during the light phase between 10 a.m. and 4 p.m., in a sound‐attenuated behavior testing room. The mice were handled by a single, unbiased experimenter. Acclimatization of mice was achieved by bringing them into the behavior testing room 1 hour before commencement of the behavioral experiments. A total of 30 mice underwent behavioral tests in three groups (10 mice per group), namely the control group, TBI group, and stem cell‐treated control group. Another group similar to the stem cell‐treated TBI group (10 mice) was taken after the final MRI.

### Open Field Test

Mice belonging to all three groups were tested one by one in an open‐field apparatus on the basis of the previously described method 15, 27. The open‐top apparatus used for this paradigm was constructed with acrylic glass of dimensions 40 × 40 cm^2^, enclosed by a 10‐cm wall. It was divided into 16 equal squares or grids, in which the 4 central grids were considered as the center and the rest the periphery. The behavioral paradigms were scored for a period of 5 minutes. The distance traveled by the animals was considered in different groups to evaluate the locomotor activity. After every observation period, and before testing the next group of animals, the apparatus was cleaned with 10% isopropyl solution to remove any olfactory cues left by the previous mice. This paradigm gave an overview of the behavioral functions such as stress and anxiety levels in the stem cell‐treated TBI group as compared with the TBI group.

### Forced Swim Test

This behavioral test is a well‐established model to test the level of depression in control mice as compared with treated mice. It is performed along the lines of the previously described method 17, 33. The apparatus for this test was a cylindrical plastic jar, 30 cm in height and 10 cm in diameter. It was filled with water at room temperature up to the 20‐cm mark, before introducing the mice into it one by one. The total immobility time was noted for each mouse after it had been inside for a period of 6 minutes. The water was passed through restrainers to remove the fecal boli and other particulate matter before placing the next mouse into the jar.

### Novel Object Recognition Test

This test uses the novelty‐seeking behavior of mice, where they prefer a new object over the previously exposed old familiar object. The test was performed on the basis of the previously described method 33 and included two discrete trials. The first trial was an acquisition trial or training period, in which 5 minutes of exposure time was allotted to the mice for familiarizing themselves with two identical objects. In the second 3‐minute long test session, one familiar object was replaced by a novel object to test the animals’ recognition memory. Mice that failed to recognize the objects for more than 10 seconds were not considered in the test session. Completion of this session was followed by the return of mice to their parental cage for a 2‐hour intertrial interval. Parameters scored during the test period included time spent in approaching the object, latency to first interaction with the novel object, and the old familiar object and squares crossed in the open‐field arena. The apparatus and objects were wiped with 10% alcohol solution after the test period of each mouse. This experiment provided an overview to measure cognitive function in mice and helped to evaluate the rate of improvement in TBI mice after stem cell transplantation.

### Statistical Analysis

Statistical analysis was carried out using IBM SPSS Statistics version 20.0 (IBM, Armonk, NY, http://www.ibm.com). The change in concentration of metabolites reflected in the MRS data taken at various time points, with respect to control group mice, were compared by repeated measures of one‐way analysis of variance (ANOVA), followed by Bonferroni's multiple comparison post hoc tests. Comparison of T2* relaxation time, brain water content measurement, and behavioral test between different groups, was carried out by one‐way ANOVA followed by Bonferroni post hoc tests. The levels of significance were set at *p* ≤ .05 and *p* ≤ .001.

## Results

### Identification and Characterization of Mice MSCs

**Figure 2:**
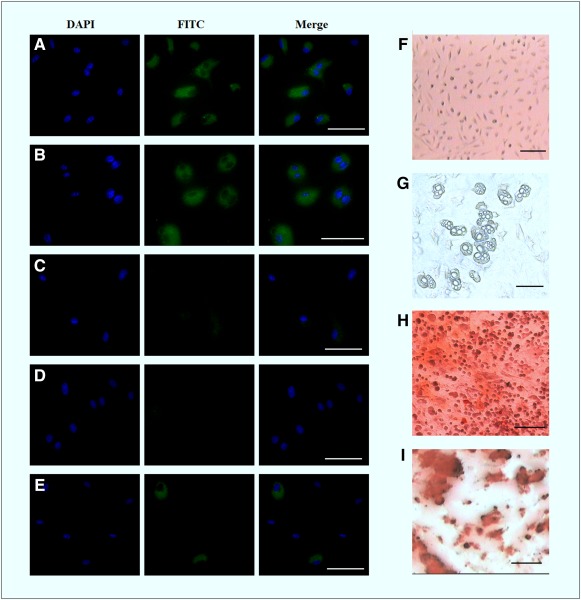
Phenotypic and differential characterization study of mMSCs. Phenotypic surface antigens’ characterization of isolated mMSCs by immunocytochemistry: Sca1 **(A)**, CD90.2 **(B)**, CD34 **(C)**, CD45**(D)**, and CD11b **(E)**. Blue fluorescent indicated Hoechst 33342 staining of nucleus, and green fluorescent indicated FITC conjugated antibodies. Scale bar: 50 µm. Osteogenic and adipogenic differentiation of mMSCs after incubation for 21 days in induction media: control MSCs **(F)**, appearance of lipid vacuoles **(G)**, alizarin staining for osteogenesis **(H)**, and Oil Red O staining for adipogenesis **(I)**. Scale bar = 100 µm. Abbreviations: DAPI, 4′,6‐diamidino‐2‐phenylindole; FITC, fluorescent isothiocyanate; mMSC, mice mesenchymal stem cell.

Stem cells isolated from mice bone marrow were found to be adherent to plastic, appearing spindle‐shape in cell culture under phase contrast microscope (Fig. 2). Immunocytochemistry results showed that the surface antigens of the isolated stem cells tested positive for mesenchymal cells (Sca‐1 and CD90.2) and negative for hematopoietic lineages (CD34 and CD45), and they showed low expression for macrophages (CD‐11b) antigen (Fig. 2A–2E). Cellular morphology exhibited a change after culturing in osteoinductive medium (spindle to cuboidal) and adipogenic medium (intercellular lipid vacuolisation) (Fig. 2F, 2G). These MSCs were found to be functionally well differentiated into osteogenic (calcium deposition) and adipogenic (lipid deposition) types in respective induction media, and appeared deep red after staining with Alizarin Red S and Oil Red O, respectively (Fig. 2H, 2I).

### Homing of Stem Cells to the Lesion Site

**Figure 3:**
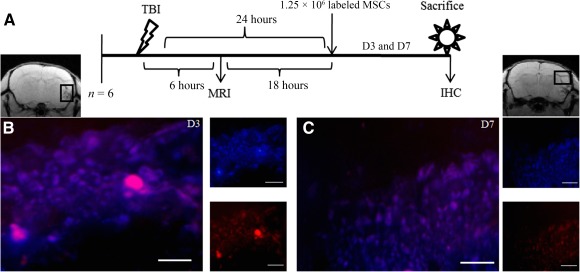
Stem cells homing to the lesion site in a brain injury model. **(A):** Systematic presentation of TBI induction, MSC administration, and sacrifice of animals for IHC. The black rectangle in MRI represents the injury site where IHC was carried out. Fluorescent figure represents stem cells homing to the site of injury on D3 **(B)** and D7 **(C)**. Red fluorescence indicates PKH26 membrane dye of administered MSCs, and blue fluorescence indicates nuclear staining dye Hoechst 33342. Scale bar = 100 µm. Abbreviations: D, day; IHC, immunohistochemistry; MRI, magnetic resonance imagining; MSC, mesenchymal stem cell; TBI, traumatic brain injury.

PKH26 labeling of MSCs was done to confirm the localization at D3 and D7 of transplantation (D4 and D8 after injury). The injured area was examined by means of fluorescent microscopy images, which suggested that transplanted PKH26‐labeled MSCs were present and had successfully reached the lesion area (Fig. 3). Additional experiments such as Y chromosome‐specific polymerase chain reaction (PCR) analysis were performed to determine whether any male‐derived cells were present at injury site after intravenous administration of MSCs isolated from male mice in TBI‐induced female mice ( supplemental online data). PCR analysis revealed the presence of male‐derived cells at injury site. The releasing factors (cytokines and chemokines) at the lesion site may have encouraged the MSCs to move chemotactically.

### T2* Map and Measurement of Relaxation Time

**Figure 4:**
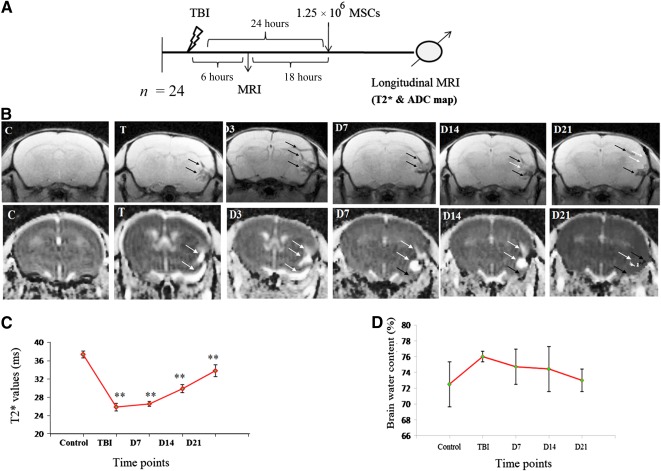
Infused MSCs increases the T2* time and decreases the edema formation at the injury site. **(A):** Schematic presentation of TBI induction, MSC transplantation and longitudinal MRI (T2* map) of animals at different time points. **(B):** Upper MRI images indicating T2* map of mouse brain at different time points using MGE_T2* sequence, and lower images indicating ADC map of mouse brain at different time points using DTI sequence, C, T, D3, D7, D14, and D21 indicated imaging after administration of MSCs in TBI mouse. **(C):** The graph presents measurement of T2* relaxation time after acquisition of T2* map at different time points. **(D):** Ex vivo measurement of total brain water content in different time points. White arrow indicates the appearance of edema (hyperintense signal), and black arrow indicates the injury area (hypointense signal) in T2* and ADC map. Data were expressed as mean ± SE of mean. The level of significance (∗, *p* ≤ .05, ∗∗, *p* ≤ .001) was shown by comparing with control using one‐way ANOVA‐Bonferroni post hoc tests. Abbreviations: ADC, apparent diffusion coefficient; ANOVA, analysis of variance; C, control; D, day; DTI, diffusion tensor imaging; MGE, multigradient echo; MRI, magnetic resonance imaging; MSC, mesenchymal stem cell; T, after TBI induction.

T2* images at respective time points, for control group (pre‐TBI mouse), TBI mouse and stem cell‐infused TBI mouse, are shown in Figure 4A–4C. After TBI, distinct hypointense signals were visible at the lesion area at 24 hours after injury and were still present on D7 (D8 after injury). A negative contrast was observed at the injury site, possibly due to the presence of microbleeding, deposition of ferritin, or tissue abnormalities (Fig. 4B). Contrast was decreased on D14 and almost disappeared on D21 (21 days after transplantation). T2* relaxation time readings were measured to observe the alteration at injury site caused by the presence of stem cells (Fig. 4C). The observed readings were significantly decreased at injury site after TBI (25.866 ± 0.829 ms, *p* < .001), as compared with control group (37.355 ± 0.767 ms). The values were also found to be significant after transplantation, on D7 (26.555 ± 0.561 ms, *p* < .001), D14 (29.844 ± 0.877 ms, *p* < .001), and D21 (33.822 ± 1.278 ms, *p* < .001). A statistically significant difference was observed between groups as determined by one‐way ANOVA (F_4,20_ = 149.158, *p* = .0001).

### ADC Map and Measurement of Brain Water Content

In the ADC map, distinct hyperintensities were observed in the injured area after TBI (Fig. 4B). Positive signal intensities persisted at D7 and decreased up to D21. It may be argued that postinjury edema formation may behave like positive contrast in ADC map, but the hyperintense signals decreased after stem cell transplantation in TBI mice. The measurement of brain water content supported the ADC map, in which the water content was higher after TBI (76.011 ± 0.695%), but it was insignificant as compared with the control (72.496% ± 2.839%). The percentage of water content was decreased at subsequent time points (D7, 74.723% ± 2.211%; D14, 74.429% ± 2.837%; D21, 72.992% ± 1.427%), implying removal of excess water content, which enhanced the recovery of injury after administration of MSCs (Fig. 4D). After statistical analysis in the brain water content study, there was an insignificant difference between groups as determined by one‐way ANOVA (F_4,10_ = 1.267, *p* = .345).

### Longitudinal Neurometabolic Changes

**Figure 5:**
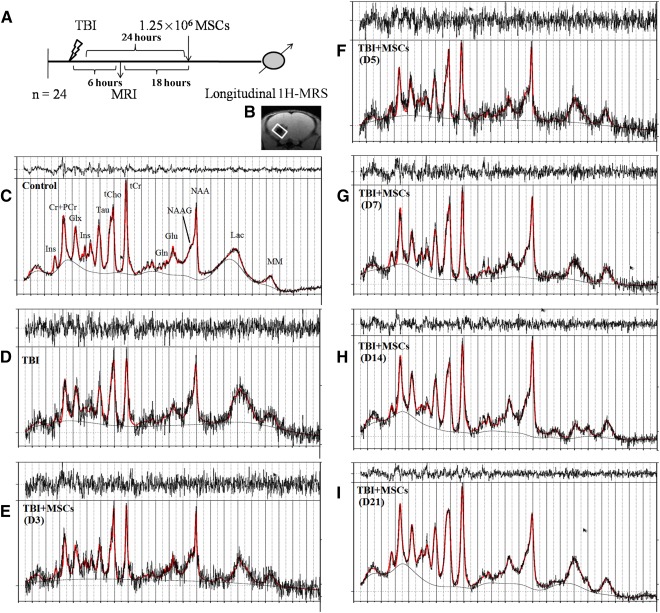
Spectra represents the neurometabolic alteration after injury and its restoration after MSC administration. **(A):** Schematic presentation of TBI induction, MSC transplantation, and acquisition of 1H‐MRS at injury area of animals at different time points. **(B):** A representation of voxel placement with volume of interest at injury site for acquisition of spectra from corresponding brain area. **(C):** Representative 1H‐MRS spectra acquired in control mouse. **(D):** Spectra acquired after TBI and after MSCs transplantation on D3 **(E)**, D5 **(F)**, D7 **(G)**, D14 **(H)**, and D21 **(I)**, with respective neurochemical peaks. The solid red line is a fit to the spectra calculated by the LC model. Abbreviations: Cr, creatine; D, day; Gln, glutamine; Glu, glutamate; Glx, glutamine complex; 1H‐MRS, proton magnetic resonance spectroscopy; Ins, *myo*‐inositol; Lac, lactate; LC, linear combination; MM, macromolecules; MRI, magnetic resonance imaging; MSC, mesenchymal stem cell; NAA, *N*‐acetylaspartate; NAAG, *N*‐acetylaspartatyl glutamate; PCr, phosphocreatine; Tau, taurine; TBI, traumatic brain injury; tCho, total choline; tCr, total creatine.

Repeated longitudinal in vivo 1H‐MRS was performed to estimate neurometabolic changes after TBI and stem cell transplantation. Schematic presentation of TBI induction, MSC transplantation, and acquisition of 1H‐MRS at injury area of animals at different time points are shown in Figure 5A. Single‐voxel MR spectra images were acquired with the voxel being positioned directly under the lesion site, inclusive of the degenerate tissue area. The placement of voxel in the anatomical T2 image is shown in Figure 5B. The detected and quantified metabolites in mouse brains included NAA at 2.008 ppm; GABA at 2.27 ppm; Glx at 2.34–2.42 ppm; tCr at 3.022 ppm and 3.98 ppm; tCho at 3.22 ppm; Tau at 3.42 ppm, and mI at 3.48–3.52 ppm. Spectra obtained from control and TBI mice brain, at different time points after stem cell transplantation, are presented in Figure 5C–I.

#### *N*‐Acetylaspartate

**Figure 6:**
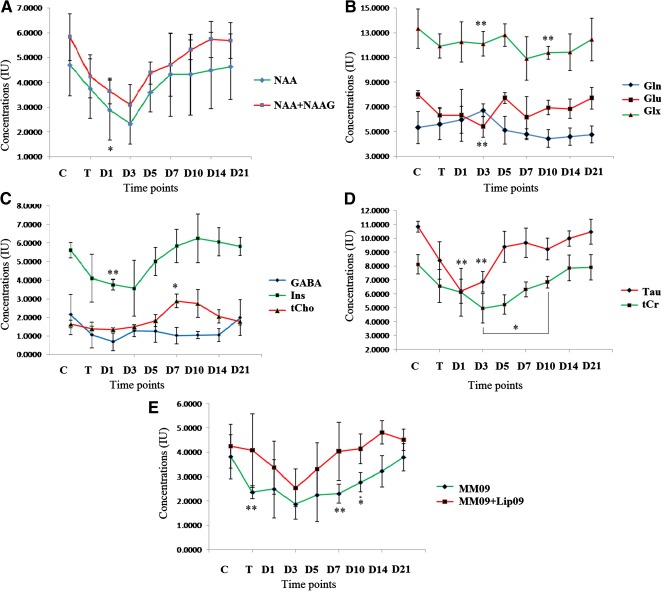
Changes in selected neurochemicals measured with 1H‐MRS. The change of metabolic concentrations of different metabolites such as NAA and NAA + NAAG **(A)**, Gln, Glu and Glx **(B)**; GABA, Ins, and tCho **(C)**; Tau and tCr **(D)**; and MM09 and MM09 + Lip09 **(E)** were evaluated with respect to different time points. Most of the metabolites decreased in concentration after TBI, but the levels remained recovered and moved toward baseline after MSC transplantation. Concentrations were expressed in institutional units and represented as mean ± SE of mean. The level of significance (∗, *p* ≤ .05, ∗∗, *p* ≤ .001) was shown by comparing with control by repeated measures of one‐way ANOVA‐Bonferroni post hoc tests. Abbreviations: ANOVA, analysis of variance; Cr, creatine; D, day; GABA, γ‐aminobutyric acid; Gln, glutamine; Glu, glutamate; Glx, glutamine complex; 1H‐MRS, proton magnetic resonance spectroscopy; Ins, *myo*‐inositol; MSC, mesenchymal stem cell; NAA, *N*‐acetylaspartate; NAAG, *N*‐acetylaspartatyl glutamate; T, after TBI induction; TBI, traumatic brain injury; tCho, total choline; Tau, taurine.

It was observed that in comparison with control, NAA decreased rapidly after injury (−20% in TBI), and reached its lowest level on D3 (−50%) (Fig. 6A). The concentration however, increased up to D21 (−1.3%) after stem cell transplantation, but no significant changes were observed at different time points in comparison with the control. Levels of total NAA (NAA + NAAG) were seen to decrease significantly on D3 (−47%, *p* = .016), maintained an increasing trend, and recovered maximally on D21 (−2.4%). The mean score for NAA and NAA + NAAG concentrations were computed by using an ANOVA with repeated measures along with Greenhouse‐Geisser correction and were found to be statistically insignificant as compared with the control, NAA (F_2.279,11.396_ = 1.895, *p* = .193), and NAA + NAAG (F_2.305,11.523_ = 3.698, *p* = .053).

#### Glu, Gln, and Glx

In contrast, neurotransmitters such as Glu and Gln followed an opposite trend: Glu reduced after TBI (−21%), and reached a significantly low limit on D3 (−33%, *p* = .009) (Fig. 6B). The concentration increased steadily and significantly on D5 (*p* = .002), D10 (*p* = .034), and D21 (*p* = .007) in comparison with its lower limit attained on D3. With respect to Gln, the concentration was significantly increased on D3 (+26.3%, *p* = .013), and exhibited inconsistency thereafter: it decreased initially and showed significant changes on D10 (*p* = .002) as compared with D3, after which it recovered to its baseline.

In the case of Glx (Glu+Gln), the concentration was found to be significantly lower on D3 (−45.5%, *p* = .005) and D10 (−27.2%, *p* = .037) and then approached the baseline. Applying the test of ANOVA with repeated measures along with Greenhouse‐Geisser correction, the mean score for Glu concentration was found to be statistically insignificant (F_1.720,8.598_ = 4.347, *p* = .054); Gln and Glx, however, showed significant differences (F_2.050,10.252_ = 4.945, *p* = .031), and (F_2.446,12.230_ = 5.038, *p* = .021), respectively.

#### γ‐Aminobutyric Acid

Levels of other neurotransmitters such as GABA fell after TBI and did not follow any consistent pattern thereafter (Fig. 6C). No statistical significance was observed on repeated measures of Greenhouse‐Geisser correction (F_2.135,10.676_ = 3.253, *p* = .077).

#### GPC + PCh

A differential change was noticed in GPC + PCh concentration after TBI (Fig. 6C), which came down (−15%) and attained maximum reduction on D1 (−18%), thereafter showing a sharp increase until D7 (+76%, *p* = .017), and then it resolved toward its normal level by D21 (+8.8%). The test of ANOVA with repeated measures with Greenhouse‐Geisser correction yielded a mean score for GPC + PCh concentration that was statistically significant (F_1.789,8.944_ = 14.338, *p* = .002).

#### Total Cr

The concentration of total Cr (CR + PCr) after TBI, and at different time points after stem cell transplantation, was variable (Fig. 6D). It was therefore not used as a normalizing factor while comparing different metabolites. These levels decreased after TBI (−19.3%), reached maximum lowest level on D3 (−39%), and then increased sharply to achieve baseline by D21. There was a significant change on D10 (*p* = .049) as compared with D3. Statistical significance was obtained in tCr after repeated measures with Greenhouse‐Geisser correction (F_2.589,12.943_ = 8.430, *p* = .003).

#### Tau and Ins

Tau levels reduced after TBI and were found to be significantly different on D1 itself (−42.8%, *p* = .002) and D3 (−36.7%, *p* = .002) (Fig. 6D). Recovery toward baseline levels was seen after stem cell transplantation. Ins concentration came down after TBI and was significantly different on D1 (−33%, *p* = .006). It increased steeply up to D10 (+11.3%) and subsequently returned to its baseline concentration. Mean scores for Tau and Ins concentrations were statistically significant (F_2.918,14.591_ = 21.958, *p* = .0001, and F_2.301,11.506_ = 6.831, *p* = .009, respectively).

#### Macromolecules

Levels of macromolecules and lipid residue containing macromolecules descended after TBI (−40 to −50%), and recovered completely after MSC transplantation. Mean scores for MM concentration were statistically insignificant based on ANOVA with repeated measures (F_1.579,7.894_ = 2.375, *p* = .160).

### Assessment of Behavioral Outcomes

**Figure 7:**
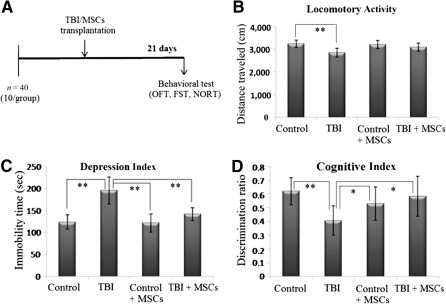
Improved functional outcomes in injury mice after MSC infusion. **(A):** Schematic presentation of behavioral test (OFT, FST, and NORT) in different groups. Behavioral test such as locomotor activity **(B)**, depression index **(C)**, and cognitive index **(D)** were carried out in the control group, TBI group, MSC‐induced control group, and MSC‐induced TBI group. Data were expressed as mean ± SE of mean. The level of significance (∗, *p* ≤ .05, ∗∗, *p* ≤ .001) was shown by multiple comparison with one‐way ANOVA‐Bonferroni post hoc tests. Abbreviations: ANOVA, analysis of variance; FST, forced swim test; MSC, mesenchymal stem cell; NORT, novel object recognition test; OFT, open field test; TBI, traumatic brain injury.

Schematic presentations of behavioral tests (OFT, FST, and NORT) in different groups are shown in Figure 7A.

#### Open Field Test

The open field test (OFT) was carried out to assess anxiety levels in mice as effected by TBI and MSC transplantation. The test involved measuring the total distance traveled by different groups of mice in an open field chamber (Fig. 7B). The distance traveled by the TBI group (2,845 ± 192 cm, *p* = .001) was significantly less than the control group (3241 ± 160 cm), and the locomotor activity was seen to increase in the stem cell‐treated TBI group (3092 ± 175 cm). Statistical analysis of OFT results showed that there was a significant difference between groups as determined by one‐way ANOVA (F_3,28_ = 8.188, *p* < .001).

#### The Forced Swim Test

The forced swim test (FST) assessed the depression index of the animals by measuring total immobility time (Fig. 7C). The immobility time was significantly increased in TBI group (195.4 ± 30.8 seconds) as compared with control group (123.5 ± 16.4 seconds), whereas the stem cell‐transplanted TBI group showed a significant decrease and improvement in the immobility time duration (141.5 ± 14.88 seconds). Statistical analysis of FST results indicated that there was a significant difference between groups as determined by one‐way ANOVA (F_3,28_ = 20.402, *p* < .001).

#### Novel Object Recognition Test

The novel object recognition test (NORT) gave an indication about stress‐induced changes in the short‐term memory functions of cognitive tasks (Fig. 7D). Discrimination ratio is the ratio between time spent with a novel object and total time spent with novel plus familiar objects. A low discrimination ratio implied that the mice spent more time with the old object than with the novel object, thereby suggesting that short‐term memory loss had taken place. The TBI group showed comparatively lower discrimination ratio values (0.406 ± 0.105, *p* = .007) than the control group (0.62 ± 0.098) and recorded an increase after stem cell treatment (stem cell‐treated TBI group recorded 0.583 ± 0.145). Statistical analysis of the NORT findings revealed a significant difference between groups as determined by one‐way ANOVA (F_3,28_ = 4.931, *p* = .007).

## Discussion

MSCs have the potential to differentiate and proliferate into various mesodermal tissue lineages in a culture medium. Furthermore, MSCs have tremendous therapeutic potential in a variety of injuries and neurodegenerative diseases. In addition to their immunoregulatory behavior, tissue response to the infused stem cells would be primarily based on paracrine mechanisms by the release of trophic and immunomodulatory factors. The present study comprised repeated in vivo assessments of neurometabolites at the brain lesion area, after transplantation of stem cells. The criteria examined included postinjury edema formation time (by visualizing in ADC map) and measurement of brain water content. These results were corroborated with MRI and 1H‐MRS findings, as well as by behavioral assessment outcomes, to evaluate the therapeutic potential of stem cell administration at the injury site.

Several reports from research literature have illustrated the homing of systemically implanted MSCs to the site of injury. One group of researchers performed a longitudinal imaging study coupled with immunohistochemistry experiments to demonstrate the successful homing of stem cells to sites of inflammatory brain injury, after intracerebral and intravenous administration 11. Another report established the migration of stem cells to the injured spinal cord in a stromal cell‐derived factor 1‐ and growth factor‐dependent manner 34. Francois et al. performed the engraftment of MSCs in radiation injury mouse model and demonstrated that the level of engraftment increased not only at the radiation induced site, but also in all other body parts 35.

The present study involved transplantation of PKH26‐labeled MSCs in traumatic brain injury mice, and validation of the successful homing of stem cells by IHC analysis (Fig. 3) and Y chromosome‐specific PCR analysis ( supplemental online Fig. 1). As mentioned earlier, stem cells have a homing response to injured tissues and organs, mediated by interactions of cytokine/chemokine receptors expressed on the stem cells, and cytokine/chemokines secreted by the injured tissue. Many factors influence this migration and homing process: age and passage number of the cells, culture conditions, expression of receptors/adhesion molecules, and, finally, the route of administration 9, 10. Taking all the responsive factors into consideration, bone marrow‐derived MSCs (passage 4) were administered intravenously in mouse tail vein after 24 hours of TBI induction and proved homing to be successful, as shown by IHC and PCR analysis.

Marmarou's TBI model in mice reproduces a diffuse type of edema that affects mainly glial cells but not neurons 1, 26, 31. ADC map findings of the present study showed the appearance of positive signal intensity at injury site after TBI, which may be attributed to edema formation (Fig. 4B). The pattern of edema may be either cytotoxic or vasogenic in character, depending on cell swelling, blood‐brain barrier dysfunction, and tissue disruption along with transfer of water and electrolytes 19. The nature of edema was ascertained using MRI technique (T2 relaxation time in association with ADC map): low relaxation time and low ADC values indicate cytotoxic edema, which appeared just after injury, whereas high T2 and ADC values indicate vasogenic edema 36, 37. In the present undertaking, a decrease in T2* relaxation time (Fig. 4C) was noted, as well as an increase in water content at the lesion area, suggesting appearance of cytotoxic edema (Fig. 4D). This observation was in accordance with our MRI findings (ADC map).

These results were also in agreement with previous research literature reports, in which MRI was suggested as a more sensitive means for detecting brain edema at the lesion site after experimental TBI. Yet another report utilized T2 and ADC imaging to demonstrate the formation of ischemia insults only 3.5 hours after the onset of ischemia 26. Corroboration of our findings from imaging, relaxometry, and brain water content studies demonstrated without doubt the elevation of T2* values and reduction of water content on D14 and D21 after stem cells administration in TBI mice (Fig. 4). This may be explained as the therapeutic effect of administered stem cells, which enhanced the rejuvenation of injured tissue and restoration of water content to normal levels.

Marmarou's model is more applicable for examining the progression of global neurometabolic changes, rather than studying induced focal cerebral injury 38. The present investigation evaluated the neurometabolic alterations in the TBI site, in response to TBI, and after stem cell transplantation in the same mice, in a longitudinal time course of 21 days. MRS provided the complete in vivo metabolic information of cells at injury site, which reflected the metabolic contribution in pathological mechanisms (Fig. 5). The spectra quality of TBI group up to D5 was not as good as others, which may be due to brain tissue degeneration and formation of edema at injury site. Thus, it is difficult to find high‐quality spectra (high signal‐to‐noise ratio) from the injury site. After D5, we had high‐quality spectra, which may be due to the repairing capability of transplanted MSCs.

For MRS reproducibility, metabolite levels from each voxel in the volume of interest were quantified using the LC model analysis algorithm of all metabolites in each time point. %SD obtained from the LC model was used as a filter in MRS data reproducibility ( supplemental online Table 1). We have set a threshold of 20% estimated SD of the metabolite fit, as output from the LC model, to ensure reliable metabolite quantitation. However, the %SD of all metabolites in each time point was found to be <20%, which indicated the reproducibility of MRS data ( supplemental online Table 1).

NAA is an important multifunctional amino acid, mainly synthesized in the mitochondria of neurons. Decreased NAA concentration may reflect mitochondrial dysfunction as well as neuronal stress or loss 20. An earlier study correlated NAA levels with the severity of injury and showed the same time course as the ATP modifications 39. Therefore, it follows that decreased NAA levels may indicate impaired energy homeostasis. Many previous studies (and the present one) showed decrease in postbrain injury NAA levels, with the reduction varying from 15% to 70% 39. Moreover, NAA is a neuronal marker whose level on 1H‐MRS correlates with histological analysis. The present experiment found a considerable decrease in NAA after 24 hours of TBI (−20%), which continued up to D3 (−50.2%) but subsequently approached baseline (Fig. 6). This could be due to tissue repair and improvement in mitochondrial function, brought about by the transplanted stem cells.

A number of metabolites such as Glu, Gln, GABA, and *N*‐acetylaspartatyl glutamate (NAAG) play an important role in functional neurotransmission, which is believed to be completed through the Glu‐Gln cycle 40. During neurotransmission, Glu is released from the presynaptic terminals and converted to Gln via the Gln synthetase pathway in astrocytes. The newly formed Gln travels back to the neuron, where it is reconverted to Glu by a mitochondrial enzyme, namely phosphate‐dependent glutaminase. The Glu undergoes decarboxylation inside the neuron and forms GABA catalyzed by glutamic acid decarboxylase. TBI induces a rapid neuronal depolarization and release of the excitatory neurotransmitter Glu, which initiates neuronal damage. To compensate for Glu concentration and reduce damage, astrocytes take up the Glu and convert it to Gln by the Glu pathway 20. Results from the present study also authenticated decreased Glu (−22%) and increased Gln (+5%) concentrations after TBI. These types of Glx (Glu+Gln) alterations have also been reported in cerebral ischemia and mild TBI (Fig. 6). The concentrations of Glu and Gln approach baseline on D21 after stem cell infusion, indicating restoration of mitochondrial function by differentiation into healthy neuronal cells.

Metabolites such as Tau, Ins, NAA, and Glu serve as cellular osmolytes. In the present study, postinjury edema caused brain cells to emanate cellular osmolytes to maintain volume homeostatis. Earlier reports associate the decrease in Tau and Ins with edema and NAA and suggest that Glu may take part in homeostatic response 14. Organic osmolytes such as Tau and Ins were believed to be primarily located in glia and absent in neurons 41. Cytotoxic injury can occur after TBI and is commonly linked to focal ischemia and stroke. One study has shown diminished transportation of Ins after stroke 42. A rise in intracellular electrolytes and accumulation of osmotically active metabolites would normally be balanced by Ins efflux. Disturbance in bioenergetics after injury brings down the requirement for the volume‐sensitive organic osmolyte anion channel, mI efflux; these changes are attributed to osmolality change in glia 20, 42. In keeping with the described reports, it was found this experiment elicited a significant decrease in postinjury levels of Tau (−42%) and Ins (−33%), and their approach toward baseline after stem cells transplantation, which is indicative of glial proliferation and restoration of local tissue osmolality.

Any damage to neural cells produces membrane phospholipid (tCho) from the plasma membrane. Increased levels of this substance have been reported in human TBI, whereas the level is decreased in animal models 42. The present study observed a mixed response, with a considerable lowering of tCho concentration after TBI (−15%), and a significant upswing on D7 (+76%). The reduction of tCho after injury may have been caused by membrane degradation at the lesion area, which was evident from histopathological analysis 19, 20, 43. The infused stem cells, after homing to the lesion site, may differentiate into neuronal cells, depending on the niche, signaling processes, and replacement of injured cells, thus indicating restoration of tCho concentration 18, 19, 44.

Intracellular Cr and PCr act as energy intermediates, and assume a critical role in cellular bioenergetics and function 19, 20. Many investigators have assumed tCr as relatively constant and, therefore, use it as a reference for 1H‐MRS studies under different physiological conditions 14, 20. In the present study, a dramatic decrease in tCr concentration was observed after TBI (−19%), which persisted until D3 (−39%). Consequently, tCr could not be considered as a reference metabolite, and results were supported by other evidence (Fig. 6).

In consistency with the 1H‐MRS findings of this study, improvement in behavioral outcomes was observed after stem cell transplantation in TBI mice (Fig. 7). The anxiety level, depression index, and cognitive index were assessed in control group, TBI group, and stem cell‐treated control and TBI group. There was a significant decrease in locomotor activity, depression index, and cognitive index after TBI, possibly due to postinjury neural dysfunction and degeneration of neural tissue in the hippocampus region 5, 27. The stem cell‐treated TBI group displayed enhanced behavioral activity, illustrating the replacement of damaged tissue with new functional neural tissue by transplanted MSCs 15, 17.

TBI is a global health problem, and there is no clinical treatment available to date. Most of the posttraumatic neurodegeneration occurs after primary injury and proceeds in a cascade of pathochemical and pathophysiological mechanisms 20, 45. Researchers are focusing increasingly on the use of regenerative medicine (stem cell‐based therapies) as a tool for generating new tissues at the injury site. To achieve this end, a number of clinical trials are being conducted in the field of cell based tissue injury 9, 27. MSCs are hypoimmunogenic and possess the additional ability of secreting and inducing the expression of trophic and growth factors in parenchymal cells 11, 17. After homing to the lesion area, complete response of MSCs is determined by its niche, consisting of surrounding cells and the releasing factors of the stem cells, which may exhibit either autocrine or paracrine actions 10, 12. The synergistic interaction between stem cells and paracrine niche molecules at the injury site decide their tisue‐forming effective regenerative response, known as the induced stem cell potency, which is cell number and niche dependent. The combination of a perfect cell administration and significant stem cell homing may enhance neuronal regeneration and improve functional outcomes.

## Conclusion

It may be reiterated that the application of allogenic cultured MSCs forms a potential therapeutic strategy for limiting the progression of secondary injury and improving functional outcome. The value of a multimodal system of evaluation (using both MRI and 1H‐MRS) for providing detailed information in TBI mice before and after MSCs transplantation is unquestionable. The presence of cytotoxic edema was confirmed by the T2* relaxation study and brain water content measurement after injury. Successful monitoring of the lesion site after MSCs transplantation was possible using in vivo 1H‐MRS longitudinally up to 21 days. Gradual recovery of altered metabolite concentrations in the lesion area was observed in a time‐dependent manner. Another positive outcome of MSCs transplantation was the possible neurogenesis, change in plasticity, and recovery of neurometabolites as a result of a stimulation of synergistic effect and enhanced behavioral activity. It may be said that this unique type of cell based therapy may serve as a restorative medicine that can be used in the clinical management of innumerable challenging medical conditions.

## Author Contributions

S.K.M.: conception and design, collection and assembly of data, data analysis and interpretation, manuscript writing; P.R.: data analysis and interpretation, revision of manuscript; S.K. and G.G.: supervision of the experimental work, provision of study materials, final approval of manuscript.

## Disclosure of Potential Conflicts of Interest

S.K.M. has uncompensated employment, intellectual property rights, stock options, honoraria, and research funding, is a consultant, has given expert testimony, and other. S.K. has uncompensated research funding. G.G. has uncompensated employment, intellectual property rights, honoraria, research funding, and stock options, is a consultant, and has given expert testimony. The other author indicated no financial relationships.

## Supporting information

Supporting Information
