# Supplementary material for: Therapeutic Prospective of Infused Allogenic Cultured Mesenchymal Stem Cells in Traumatic Brain Injury Mice: A Longitudinal Proton Magnetic Resonance Spectroscopy Assessment
Source: Stem Cells Transl Med. 2016 Aug 8;6(1):316–29. doi: 10.5966/sctm.2016-0087 (PMC5442758; doi:10.5966/sctm.2016-0087)
Supplement: Supplementary file 1 — Supporting Information [file SCT3-6-316-s001.pdf]

Supplemental Information – Khushu et al

Supplementary material S1

PCR analysis:

In this experiment, isolated MSCs (1.25 x 10^6 cells) from male Balb/c mice were injected intravenously in traumatic brain injury induced female mice (n=3). After seven days of post-transplantation, the mice were sacrificed and the site of injury was dissected out for DNA extraction. Y-chromosome specific PCR was performed to determine whether any male derived cells were present at injury site. PCR amplification also carried out in male mice (positive control) and female mice (negative control). The oligonucleotide primers were as follows: *syr* locus (F: 5'-CTG CTG TGA ACA GAC ACT AC-3'; R: 5'-GAC TCC TCT GAC TTC ACT TG-3') and *GAPDH* (F: 5’-ACC ACA GTC CAT GCC ATC AC-3’; R: 5’-CAC CAC CCT GTT GCT GTA GCC-3’). PCR amplification cycling conditions: pre-incubation at 95 ^{o}C for 10 min, followed by 40 amplification cycles (95°C for 30 s, 55-68 ^{o}C gradients (depending on primers) for 30 s and 72 ^{o}C for 30 s) and a final 72 ^{o}C for 5 min. These primers amplified a DNA fragment of 722 bp and 450 bp for syr gene and GAPDH respectively.

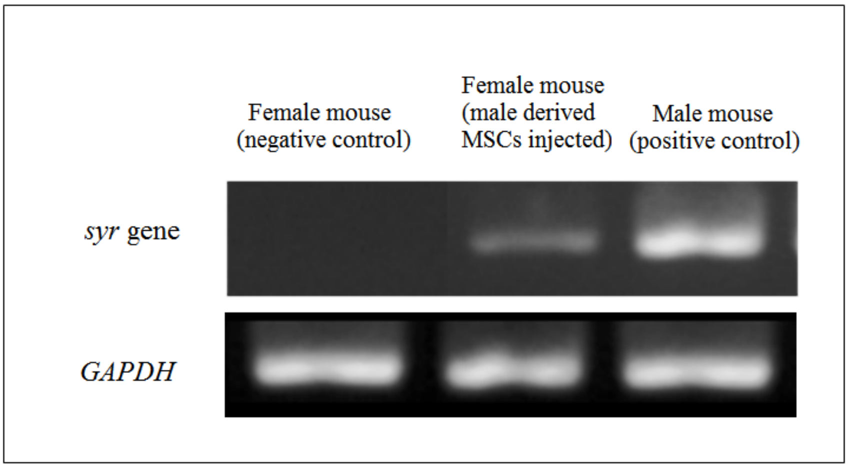

Fig. S1: Y-chromosome specific PCR: PCR products of brain tissues in MSCs treated female mouse (sample), male Balb/c mouse (positive control) and female Balb/c mouse (negative control) were analyzed on a 1.5% agarose gel. GAPDH served as an internal control.

The electrophoresis images showed the presence of male derived cells in MSCs treated mouse. The PCR analysis in association with histological fluorescent imaging has confirmed the proper homing of transplanted MSCs at the site of injury.

Supplementary Table-1

Mean standard error estimates (%SD or CRLB) generated by LCModel for each metabolite of each time point (n=6).

Metabolites	Control	TBI	DAY-1	DAY-3	DAY-5	DAY-7	DAY-10	DAY-14	DAY-21
**NAA**	9.00	14.20	17.67	5.50	9.00	11.75	11.25	8.50	9.05
**NAA+NAAG**	6.50	11.40	16.67	5.50	7.25	9.50	9.25	7.25	7.65
**Gln**	16.83	16.50	17.00	16.50	17.75	15.38	15.50	16.25	16.30
**Glu**	9.17	15.90	17.00	10.00	9.75	12.88	13.00	10.25	10.80
**Glx (Gln+Glu)**	7.85	12.60	15.00	11.25	9.50	11.63	13.50	9.50	10.30
**GABA**	15.33	14.10	12.00	15.25	17.75	16.75	15.25	15.50	16.45
**Ins**	8.33	18.30	17.33	7.25	10.00	8.88	10.25	7.50	8.05
**tCho**	7.00	9.30	12.67	6.25	6.75	7.38	6.75	5.75	5.95
**Tau**	5.00	7.90	11.33	5.00	5.75	6.38	6.75	5.00	5.35
**tCr**	4.00	6.10	9.67	4.00	4.75	4.88	5.00	4.00	4.20
**MM09**	18.17	17.90	16.33	16.25	16.00	15.75	16.00	17.50	16.80
**MM09+Lip09**	16.00	17.80	17.00	14.25	16.50	16.63	13.75	14.50	14.35
